# Investigation of the Relationship Between Brown HT Dye Exposure and Mammary Tumor Development in Female Rats: An Assessment of the Potential Risk of Breast Cancer

**DOI:** 10.7759/cureus.73351

**Published:** 2024-11-09

**Authors:** T. M. Tawabul Islam, Nirmal Chandra Mahat, Ivvala Anand Shaker, Sheikh Arafat Rahman, Md. Humayan Kabir, Mustakin Ahmed Shohel, Md. Kamruzzaman, Abul Kashem Tang

**Affiliations:** 1 Department of Food and Nutrition, Faculty of Applied Science, Parul University, Vadodara, IND; 2 Department of Applied Nutrition and Food Technology, Islamic University, Kushtia, BGD; 3 Department of Biochemistry, Swaminarayan Institute of Medical Sciences and Research, Swaminarayan University, Shree Swaminarayan Vishvamangal Gurukul, Kalol, IND

**Keywords:** alpha-fetoprotein, breast cancer, brown ht, dcis, mammary tumor

## Abstract

Background: Azo dyes featuring one (monoazo) or several intramolecular NQN bonds are utilized in the food, pharmaceutical, and textile industries. The food azo dye chocolate brown HT (E155) adversely affects hepatic and renal function upon prolonged consumption. This study aimed to assess the carcinogenic potential of E155 in the development of mammary tumors and breast cancer.

Methods: A total of 20 female Long-Evans rats (eight to nine weeks old) were randomly assigned to five groups, each consisting of four rats. The control (female control) group received a regular diet, whereas the positive control (female positive control) group received 7,12-dimethylbenz(a)anthracene. The remaining three groups received 200, 400, or 600 mg/kg body weight (BW)/day E155 for 40 weeks. Tumor development, BW, and biochemical, hematological, and histological data were monitored.

Results: BW decreased significantly with increasing dosages in the female moderate dose (FMD) group. Blood counts indicated potential microcytic anemia and inflammation in the treatment groups, especially in the female high-dose (FHD) group. E155 dose-dependently impaired renal function and increased blood creatinine and uric acid levels. Elevated serum glutamic pyruvic transaminase (SGPT) and serum glutamic-oxaloacetic transaminase levels indicate abnormal liver function. FHD animals had more tumors and larger sizes. Higher alpha-fetoprotein (AFP) and cancer antigen levels were detected even at low doses. Histopathological analysis revealed that E155 causes mammary gland fibroadenomas, ductal carcinoma in situ, and hyperplasia. It also causes circular layer granulomas, fibrosis, and crypt abscesses in the intestines of FMD and FHD.

Conclusion: The current study suggests that prolonged exposure to E155 may result in a higher incidence of mammary tumors, indicating an elevated risk for the onset of breast cancer.

## Introduction

Azo dyes are a group of chemicals that have been extensively examined because they are commonly used in textiles, food, cosmetics, etc., and pose a possible risk to health. Azo dyes have been shown to contain a nitrogen-nitrogen (-N=N-) double bond in their structure, and these compounds are metabolically activated into highly toxic and reactive metabolites that interact with DNA, causing mutations [1]. The extensive use of azo dyes requires considerable knowledge of their cancerous potential to ascertain the future health consequences associated with exposure to these substances.

Several azo dyes have been found to be carcinogenic and mutagenic agents capable of causing allergic reactions [2]. Typically, the toxicity of a compound increases with the number of benzene rings present. The carcinogenic effects of azo dyes depend on their molecular structure and degradation mechanism. After their breakdown, most of these dyes contain diverse aromatic amines that may also act as carcinogens [2]. Some azo dyes are not cleaved into aromatic amines but still become carcinogenic, whereas others are cleaved through their cleavage products, such as benzidine. Benzidine can induce several types of tumors in both humans and animals. In addition, p-phenylenediamine, another constituent of azo dyes, is an allergen. These dyes can be nonbiologically reduced by human intestinal microflora, skin microflora, and environmental microorganisms and, to a lesser extent, by human liver azo reductases [1].

For more than a century, these colorants have been recognized for their brightness and longevity in synthetic materials, including textiles, food additives, cosmetics, and pharmaceuticals. It is primarily used for food coloring, especially in baked goods, confectionaries, and beverages. Some countries have banned these chemicals in food, such as Canada, Japan, and Norway, while others have restricted use [3], although unregulated in developing countries like Bangladesh. The extensive use of azo dyes has raised questions about their safety because they easily convert into aromatic amines, which are carcinogenic compounds and play a part in tumorigenesis [2].

Azo dyes can cause mammary tumors in several ways. One possible mechanism is oxidative stress, which is caused by the azo dye, leading to DNA damage and eventual malignant transformation of different organs, such as the breast. Another possible mechanism is mitochondrial dysfunction, which is triggered by azo dye by inhibiting mitochondrial enzymes [4]. The mammary gland is responsive to carcinogenic insults because of its rapid cell division and hormonal activation; hence, it is used for chemical carcinogenesis. One major well-characterized detoxification pathway involves the reduction of azo bonds to release aromatic amines, which can be further metabolized to reactive forms by cytochrome P450 enzymes. These intermediates can then form covalent bonds with DNA molecules, thus forming DNA adducts that can cause mutations and result in carcinogenesis. Moreover, the ability of azo dyes to induce oxidative stress can induce the formation of reactive oxygen species (ROS) and subsequent injury to cellular components such as lipids, proteins, and nucleic acids [5].

In addition to oxidative stress, inflammation is an important factor in developing and progressing azo-dye-induced mammary tumors. Initially, chronic inflammation was shown to have protumor effects due to cytokine release and the generation of growth factors and other inflammatory mediators. They can stimulate cell division, suppress cell death, and stimulate new blood vessel formation, all of which help in cancer growth and spread. Research has also revealed that azo dyes can induce chronic inflammation in the mammary gland, thereby augmenting the process of tumorigenesis [6]. Clinical chemistry results revealed elevated liver enzymes, suggesting that liver tissue may have been compromised and that metabolic pathways may have been affected [7]. Hematological analyses have revealed alterations in blood parameters, suggesting systemic toxicity and immune dysfunction [8]. Histopathological analysis of the mammary tumors supported the idea that they were carcinomas, as the tissues presented features such as increased cell density, enlargement of nuclei, and invasion of the tumors into the surrounding tissues. These findings indicate that these investigations offer primary data on the malignancy of tumors caused by azo dye exposure [9].

Chocolate brown HT (E155), or brown HT, is a synthetic bis-azo dye that is frequently used as a food coloring additive. This dye is predominantly utilized to improve the coloration of various food products such as chocolate cakes, biscuits, baked goods, ice cream, puddings, and sauces [10]. Several studies have reported that brown HT is associated with many health problems, including hepatorenal abnormalities, reductions in organ and body weight (BW), and detrimental effects on the gastrointestinal (GI) tract [10-12]. No prior study has reported the impact of E155 on the occurrence and progression of breast tumors. This study was designed to investigate the incidence and development of mammary tumors in female Long-Evans rats following long-term administration of the azo dye chocolate brown HT due to the close association of mammary tumors with breast cancer onset and the previously reported carcinogenic properties of azo dyes.

## Materials and methods

Study area

This research was conducted at the Department of Applied Nutrition and Food Technology, Islamic University, Kushtia, Bangladesh, where the primary experimental procedures were performed. Biochemical analyses were performed at the doctor's laboratory in Kushtia to ensure precision in the metabolic profiling of the subjects. Histological examinations, crucial for understanding tissue-level changes, were meticulously carried out in the anatomical laboratory at the National Medical College in Dhaka, Bangladesh. This collaborative effort across these esteemed institutions underscores the comprehensive and interdisciplinary nature of this study.

Chemicals and reagents

Chocolate brown HT (E155) is an azo dye (Echo Food Color and Aroma Ltd., Raypara, Bangladesh) used as a food-coloring agent. 7,12-Dimethylbenz(a)anthracene (DMBA) was sourced from Sigma-Aldrich (St. Louis, MO) as a carcinogenic compound for experimental purposes. Uric liquor was obtained from Human GmbH (Wiesbaden, Germany) for biochemical analysis, whereas creatinine liquor was procured from DiaSys Diagnostic Systems GmbH (Holzheim, Germany). The enzyme activities of SGOT and SGPT were assessed in the Chronolab AG (Zug, Switzerland) and Tulip Group (Gurugram, India), respectively. Direct bilirubin measurements were conducted using reagents from Randox (Crumlin, United Kingdom). Lipid profiles were evaluated using cholesterol liquicolor and triglyceride liquicolor from Biocon Diagnostics (Germany) and DiaSys Diagnostic Systems GmbH, respectively, for LDL cholesterol. Finally, reagents for histological analysis were obtained from Biolab Diagnostics (I) Pvt. Ltd. (Mumbai, India).

Animal grouping and experimental design

The Ethics Committee of Islamic University (reference no.: FBS/ERC/IU-2021/05) approved all the experimental protocols in accordance with the guidelines outlined in the "Guide for the Care and Use of Laboratory Animals" [13]. Virgin female Long-Evans rats, aged eight to nine weeks, were acquired from the International Centre for Diarrheal Disease Research, Bangladesh (Dhaka, Bangladesh) and kept in separate housing units under controlled temperature settings (23°C ± 1°C) with a 12-hour light/dark cycle. Every rat was given a daily ration of 12 g of cacks made from different concentrations of brown HT chocolate. The sample size was selected through power analysis [14], estimating 16 female Long-Evans rats for 0.80 power at a significance level of 0.05. To accommodate for heterogeneity and possible attrition, the sample size was increased to 20, assigning four female rats to each treatment group. The study consisted of five groups: a control group (female control, FC), a positive control group (female positive control, FPC), and three treatment groups (female low dose, FLD; female moderate dose, FMD; and female high dose, FHD) that received doses of 200, 400, and 600 mg/kg BW brown HT, respectively. FC was used for female rats that were not fed brown HT and comprised the control group. The positive control group (FPC) was not administered brown HT but received the carcinogen DMBA at a dose of 3 mg/kg BW. The treatment groups were divided according to the dosage of brown HT and were administered a low dose of FLD at 200 mg/kg BW/day, a moderate dose of FMD at 400 mg/kg BW/day, and a high dose of FHD at 600 mg/kg BW/day. The doses were justified based on the nonobserved adverse effect level of 143 mg/kg BW/day defined by the Joint Food and Agriculture Organization/World Health Organization Expert Committee on Food Additives [3]. As no studies have indicated any occurrence of malignancy due to short-term administration of chocolate brown HT, this trial was longitudinal, spanning 40 weeks, during which the effects of various doses were investigated in rats.

Blood sampling

Finally, to assess the therapeutic effectiveness of the dye, blood was collected from each rat 40 weeks (nine months) after the experiment via cardiac puncture. Subsequently, the blood samples were allowed to clot at room temperature and centrifuged at 3,000 rpm for 10 minutes to precipitate serum. Serum was used to determine biochemical parameters. To ensure the accuracy of the results, serum samples were assayed using fully automatic biochemical analyzers and commercial test kits [15].

Hematological, biochemical, and biomarker analyses

A broad panel of hematologic tests was thoroughly examined using the modern automatic Sysmex XN-1000™ Hematology Analyzer developed by the Sysmex Corporation (Kobe, Japan). This analyzer was chosen for its accuracy and reliability in rendering abreast of hematological analysis. The evaluated parameters included the following: full blood count, red blood cell (RBC) count, white blood cell (WBC) count, hemoglobin (Hb) level, mean corpuscular volume (MCV), mean corpuscular hemoglobin (MCH) level, mean corpuscular hemoglobin concentration (MCHC), and platelet, neutrophil, lymphocyte (LY), monocyte, eosinophil, and reticulocyte counts. A HITACHI Cobas c 311 analyzer (Roche Diagnostics Ltd., Shanghai, China) was used to systematically assess numerous biochemical aspects. The presence of microcytic anemia was determined using the standard cutoff described elsewhere [16,17]. The chosen analyzer, known for its accuracy and dependability, was used to assess the following parameters: serum cholesterol, high-density lipoprotein, low-density lipoprotein, triglyceride, serum creatinine, serum glutamic-oxaloacetic transaminase (SGOT/aspartate aminotransferase), serum glutamic pyruvate transaminase (SGPT/ALT), serum uric acid, and serum bilirubin. A fully automated clinical chemistry analyzer, the HITACHI cobas e 411 analyzers (Roche Diagnostics Ltd.), was used to quantify the measured amount of alpha-fetoprotein (AFP) together with the breast cancer biomarker cancer antigen (CA15-3) in the serum. An enzyme-linked immunosorbent assay was performed according to the manufacturer's instructions to provide reliable and accurate measurements of effective biomarkers. The addition of AFP and CA15-3 values provided valuable information regarding the status of the cited cancers in individuals.

Histopathological examinations

Mammary tumor tissue and small intestine samples were carefully collected and fixed in 10% neutral buffered formalin, followed by various processes of dehydration and cleaning before they were ready for embedding. The tissues were impregnated with liquefied paraffin, microembedded in paraffin blocks, and sectioned at a thickness of 5 μm. The sections were mounted on glass slides, stained with hematoxylin and eosin, and examined under a microscope. The detailed preparation method provides properly prepared histological sections of good quality, which allows evaluation of the tissue composition and recognition of changes associated with breast tumors and the small intestine [18].

Mammary tumor volume

To ensure that the actual size of the tumors that developed in the mammary gland was well understood in this study, tumors were removed after sacrificing each rat, and the size of each tumor was measured using a digital slide caliper (INSIZE 1114-200A, Taiwan). The formula for the volume of an ellipsoid was used to approximate the tumor volume: π/6 × L × W × H, where L is the length of the tumor, W is the width of the tumor, and H is the height of the tumor. This method provides a detailed calculation of tumor size, which is crucial for assessing the severity of the neoplastic process because the tumor can be considered an ellipsoid. Proper quantification of the tumor mass is essential to compare the effectiveness of treatments and monitor tumor growth trends [19].

Statistical analysis

Data are presented as mean ± standard error of the mean. The effects of various doses of brown HT were compared with those of the control and negative control groups using one-way analysis of variance, followed by Dunnett's post hoc test. All analyses were conducted using the SPSS Statistics, version 21 (IBM Corp., Armonk, NY). Statistical significance was set at a p value of <0.05.

## Results

Effects of E155 on BW

Table 1 shows the variations in BW among the groups during the 40-week observation period. In the control group (FC), BW initially increased but then plateaued after the 30th week. Compared with the control rats, the rats in the DMBA-induced positive control group (FPC) did not experience significant weight gain, and their weight began to decrease beginning in the 20th week. A similar pattern was noted in rats that were fed brown HTs, particularly in the groups that received medium and high doses. The control group showed the greatest percentage increase in BW (149.68%), whereas the medium-dose treatment group (FMD) showed the lowest increase (94.74%) after the 40th week.

**Table 1:** Effects of chocolate brown HT on body weight (g) Each value in the table is presented as mean ± standard error (n = 4) derived from one-way ANOVA followed by Dunnett's multiple comparisons. In each group, body weight after each period was compared with the initial weight in the first week (^a^p < 0.0001) The symbol (%) indicates the percentage increase in weight relative to the first week FC: female control; FPC: female positive control, induced by dimethylbenz(a)anthracene; FLD: female low dose (200 mg/kg body weight/day); FMD: female moderate dose (400 mg/kg body weight/day); FHD: female high dose (600 mg/kg body weight/day); ANOVA: analysis of variance

Week	FC (% increase)	FPC (% increase)	FLD (% increase)	FMD (% increase)	FHD (% increase)
1st	106.17 ± 3.44 (0)	107.5 ± 4.13 (0)	114.77 ± 3.27 (0)	121.89 ± 4.40 (0)	107.5 ± 5.56 (0)
10th	168.3 ± 2.50 (58.52)^a^	166.71 ± 8.11 (55.08)^a^	192.17 ± 4.74 (67.44)^a^	221.52 ± 5.48 (82.29)^a^	179.21 ± 7.98 (66.71)^a^
20th	241.6 ± 2.63 (127.56)^a^	226.46 ± 6.6 (110.66)^a^	267.33 ± 6.87 (132.93)^a^	274.26 ± 7.43 (125.75)^a^	266.46 ± 8.76 (147.87)^a^
30th	268.96 ± 1.96 (153.33)^a^	221.6 ± 2.30 (106.14)^a^	295.81 ± 4.63 (157.74)^a^	277.84 ± 4.78 (127.94)^a^	260.85 ± 10.23 (142.65)^a^
40th	265.08 ± 2.38 (149.68)^a^	216.32 ± 1.75 (101.23)^a^	251.12 ± 3.93 (118.8)^a^	237.37 ± 4.93 (94.74)^a^	233.07 ± 3.11 (116.81)^a^

Effects of E155 on the incidence of tumors

**Figure 1:**
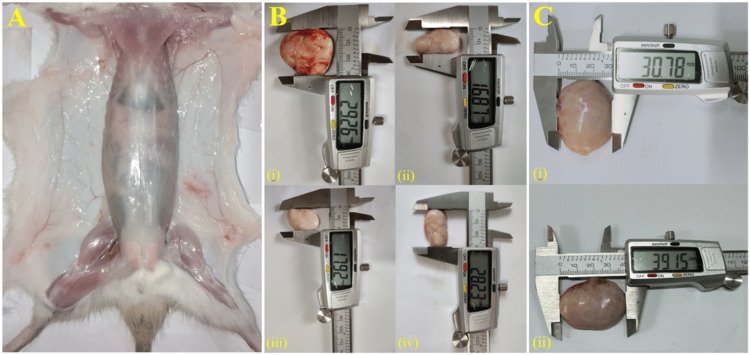
Mammary tumor image and volume are in line with the mammary glands of sacrificed rats (A) in control group (fed normal diet without E155), which has no sign of mammary gland tumor or any alteration, (B) in (i) positive control female rats treated with the carcinogen DMBA at a dose of 3 mg/kg body weight (FPC), (ii) brown HT (E155) at a dose of 200 mg/kg body weight (FLD), (iii) brown HT (E155) at a dose of 400 mg/kg body weight (FMD), and (iv) brown HT (E155) at a dose of 600 mg/kg body weight (FHD), while (C) displays the (i) width and (ii) length of the tumor DMBA: dimethylbenz(a)anthracene; FPC: female positive control; FLD: female low dose (200 mg/kg body weight/day); FMD: female moderate dose (400 mg/kg body weight/day); FHD: female high dose (600 mg/kg body weight/day)

**Figure 2:**
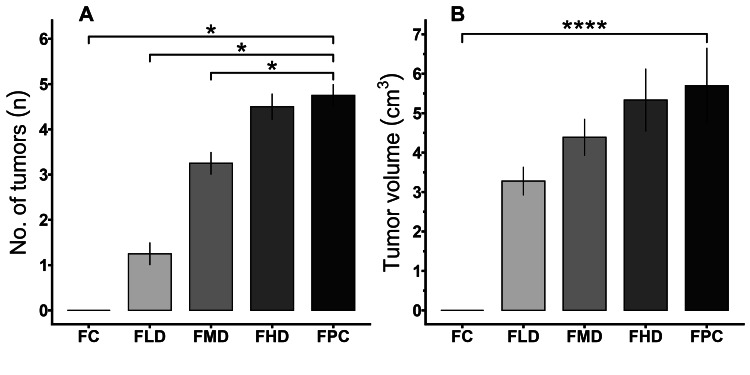
Incidence and development of tumors, (A) number of tumors, and (B) tumor volume (cubic centimeter) after prolonged ingestion of E155. Results are derived from one-way ANOVA followed by Dunnett's multiple comparisons; asterisk indicates significant difference when compared with FPC (*p< 0.05; ****p < 0.0001) FC: female control; FLD: female low dose (200 mg/kg body weight/day); FMD: female moderate dose (400 mg/kg body weight/day); FHD: female high dose (600 mg/kg body weight/day); FPC: female positive control, induced by dimethylbenz(a)anthracene; ANOVA: analysis of variance

The rats were monitored continuously to identify tumor formation. Tumor growth was initially observed in the DMBA-treated (FPC) group during the 13th week. Tumors in the treatment groups were subsequently detected the following week. Figure 1 shows an extensive illustration of the tumors, vividly showing their different sizes and unique features. After sacrifice, no tumor growth was observed in control rats (Figure 1A). A maximum of five tumors were identified in a single rat from the positive control and high-dose groups, whereas four tumors were found in a single rat from the medium-dose group, and two tumors were found in the low-dose group. Figure 2A shows the number of tumors in the different experimental groups. There were no statistically significant differences between the high-dose and positive-control groups.

Effects of E155 on tumor volume

Figure 2B depicts the mean tumor volume of the rats in several experimental groups. The positive control group (FPC) showed the highest tumor volume. The largest tumor, measuring 14.22 cm^3^, was detected in this group, surpassing all other tumors. The size of the tumors increased proportionally with dosage, although there were no significant differences between the different treatment groups fed E155.

Effects of E155 on blood count and immunological markers

Table 2 presents the comprehensive blood counts of the rats in the different groups after 40 weeks, with respect to the reference values. Noticeable abnormalities were noted in the treatment and positive control groups compared to the control groups and reference values [20-22]. The RBC count in the treatment groups decreased as the dosage increased, with no significant difference observed between the high-dose and positive control groups. The MCV level in the positive control brown HT-fed rats was reduced, indicating significant alterations in the size of RBCs. Compared with that in the control group, the percentage of hemoglobin in the high-dose treatment group was lower (p < 0.001); however, that in the high-dose treatment group was similar to that in the positive control group (p > 0.05). Hemoglobin levels in the blood, as measured by the average quantity of hemoglobin (MCH) and mean corpuscular hemoglobin concentration (MCHC), decreased substantially (p < 0.05) with increasing dosage. The LY count decreased dramatically. However, there was a considerable increase in the WBC and platelet counts. No significant differences were detected between the positive control and high-dose groups. Higher concentrations of neutrophils, monocytes, eosinophils, basophils, and reticulocytes were also observed.

**Table 2:** Effects of chocolate brown HT on complete blood count (after the 40th week) Each value in the table is presented as mean ± standard error (n = 4) derived from one-way ANOVA followed by Dunnett's multiple comparisons ^a^Significant (p < 0.05) difference when compared with FPC ^b^Significant (p < 0.05) difference when compared with FC FC: female control; FPC: female positive control, induced by dimethylbenz(a)anthracene; FLD: female low dose (200 mg/kg body weight/day); FMD: female moderate dose (400 mg/kg body weight/day); FHD: female high dose (600 mg/kg body weight/day); RBC: red blood cell; WBC: white blood cell; Hb: hemoglobin; MCV: mean corpuscular volume; MCH: mean corpuscular hemoglobin; MCHC: mean corpuscular hemoglobin concentration; ANOVA: analysis of variance

Hematological parameters	FC	FPC	FLD	FMD	FHD	Reference value
RBC (10^6^/µL)	6.68 ± 0.13^a^	4.35 ± 0.06^b^	5.53 ± 0.13^ab^	5.05 ± 0.13^ab^	4.48 ± 0.11^b^	2.9-6.8
WBC (10^3^/µL)	6.65 ± 0.20^a^	14.65 ± 0.17^b^	11.58 ± 0.28^ab^	13.15 ± 0.29^ab^	15.38 ± 0.3^b^	3.6-14.5
Hb (g/dL)	12.43 ± 0.29^a^	7.5 ± 0.15^b^	10.48 ± 0.29^ab^	9.3 ± 0.36^ab^	7.75 ± 0.12^b^	8.6-15.38
MCV (fL)	46.08 ± 0.98^a^	28.2 ± 0.48^b^	39.0 ± 0.60^ab^	33.18 ± 1.02^ab^	29.58 ± 0.29^b^	15.15-119.44
MCH (pg)	13.18 ± 0.19^a^	9.2 ± 0.11^b^	11.78 ± 0.13^ab^	10.48 ± 0.26^ab^	9.83 ± 0.19^b^	13.07-41.57
MCHC (g/dL)	30.03 ± 0.63^a^	22.23 ± 1.04^b^	28.38 ± 0.4^ab^	25.3 ± 0.42^ab^	23.65 ± 0.67^b^	21.16-95.0
Platelet (10^3^/µL)	640 ± 9.13^a^	875 ± 6.46^b^	702.5 ± 8.54^ab^	805 ± 6.46^ab^	855 ± 10.41^b^	148-615
Neutrophils (%)	16.75 ± 0.85^a^	31.25 ± 1.25^b^	23.75 ± 0.25^ab^	26.25 ± 0.48^ab^	30.0 ± 0.9^b^	13-61
Lymphocytes (%)	73.25 ± 0.85^a^	51.75 ± 1.84^b^	63.75 ± 0.25^ab^	58.25 ± 0.48^ab^	52.50 ± 0.87^b^	55-86
Monocytes (%)	8.25 ± 0.25^a^	14.75 ± 0.4^b^	10.5 ± 0.29^ab^	13.0 ± 0.4^ab^	14.5 ± 0.29^b^	6.40-10.2
Eosinophils (%)	1.50 ± 0.29	1.75 ± 0.25	1.50 ± 0.29	1.50 ± 0.29	1.75 ± 0.25	0-8
Basophils (%)	0.25 ± 0.25	0.5 ± 0.29	0.5 ± 0.29	1.0 ± 0.0	1.25 ± 0.25^b^	0-2
Reticulocyte (%)	0.83 ± 0.04^a^	2.68 ± 0.11^b^	1.74 ± 0.02^ab^	1.86 ± 0.0^ab^	2.5 ± 0.20^b^	0.55-2.5

Effects of E155 on the lipid profile and liver and renal biomarkers

Significant abnormalities in relation to the reference values in the lipid profile were identified in the treatment groups after 40 weeks (Table 3) [10,20]. The serum levels of cholesterol, LDL, and triglycerides increased proportionally with the dosage. Furthermore, the values of the high-dose group did not differ significantly from those of the positive control group. HDL levels in the treatment groups decreased. The extended use of chocolate brown HTs also affects liver function. The levels of SGOT, SGPT, and bilirubin were considerably increased, and those in the high-dose group were similar to those in the positive control group. Additionally, the treatment groups showed elevated levels of renal biomarkers. Compared with the control rats, the serum levels of creatinine and uric acid were significantly higher (p < 0.05). Nonetheless, no significant differences were observed between the FHD and positive control rats.

**Table 3:** Effects of chocolate brown HTs on the lipid profile and liver and renal function after 40th week (biochemical observations) Each value in the table is presented as mean ± standard error (n = 4) derived from one-way ANOVA followed by Dunnett's multiple comparisons ^a^Significant (p < 0.05) difference when compared with FPC ^b^Significant (p < 0.05) difference when compared with FC FC: female control; FPC: female positive control, induced by dimethylbenz(a)anthracene; FLD: female low dose (200 mg/kg body weight/day); FMD: female moderate dose (400 mg/kg body weight/day); FHD: female high dose (600 mg/kg body weight/day); SC: serum cholesterol; HDL: high-density lipoprotein; LDL: low-density lipoprotein; TG: triglyceride; SGOT: serum glutamic-oxaloacetic transaminase; SGPT: serum glutamic pyruvic transaminase; BL: bilirubin; CRT: serum creatinine; UA: serum uric acid; ANOVA: analysis of variance

Biochemical parameters	FC	FPC	FLD	FMD	FHD	Reference value
SC (mg/dL)	68.58 ± 1.24^a^	116.3 ± 1.85^b^	84.0 ± 1.85^ab^	101.18 ± 1.55^ab^	112.48 ± 1.88^b^	20.4-87.6
HDL (mg/dL)	38.2 ± 0.94^a^	16.25 ± 0.65^b^	26.6 ± 1.70^ab^	19.18 ± 0.59^ab^	17.13 ± 0.69^b^	0.2-63.3
LDL (mg/dL)	16.08 ± 0.69^a^	36.83 ± 1.06^b^	22.3 ± 1.26^ab^	27.7 ± 1.69^ab^	34.45 ± 0.89^b^	28.68-49.32
TG (mg/dL)	58.0 ± 2.84^a^	97.45 ± 0.58^b^	73.95 ± 1.91^ab^	82.2 ± 1.64^ab^	91.1 ± 2.41^b^	8.7-60.7
SGOT (IU/L)	44.10 ± 1.12^a^	84.73 ± 0.66^b^	55.15 ± 1.26^ab^	67.95 ± 1.58^ab^	80.03 ± 1.81^b^	20.8-470.2
SGPT (IU/L)	22.88 ± 1.9^a^	67.35 ± 1.08^b^	45.78 ± 1.04^ab^	57.53 ± 0.77^ab^	63.3 ± 1.01^b^	2.1-426.9
BL (mg/dL)	0.27 ± 0.01^a^	0.40 ± 0.01^b^	0.32 ± 0.01^ab^	0.36 ± 0.01^ab^	0.39 ± 0.0^b^	0.30-0.34
CRT (mg/dL)	0.63 ± 0.02^a^	1.15 ± 0.07^b^	0.79 ± 0.01^ab^	0.93 ± 0.03^ab^	1.01 ± 0.03^b^	0.2-1.2
UA (mg/dL)	2.13 ± 0.04^a^	3.92 ± 0.02^b^	2.98 ± 0.15^ab^	3.28 ± 0.13^ab^	3.76 ± 0.08^b^	2.48-2.83

Effects of E155 on cancer biomarkers

**Figure 3:**
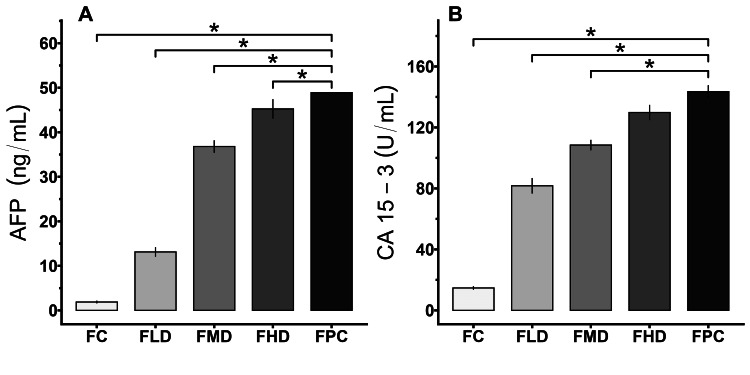
Effects of E155 on the levels of the cancer biomarkers, (A) AFP and (B) CA15-3, after 40 weeks. Results are derived from one-way ANOVA followed by Dunnett's multiple comparisons; asterisk indicate significant difference when compared with FPC (*p < 0.05) AFP: alpha-fetoprotein; FC: female control; FLD: female low dose (200 mg/kg body weight/day); FMD: female moderate dose (400 mg/kg body weight/day); FHD: female high dose (600 mg/kg body weight/day); FPC: female positive control, induced by dimethylbenz(a)anthracene; CA15-3: cancer antigen; ANOVA: analysis of variance

Compared with those of the control rats, the levels of AFP significantly increased (p < 0.05) in a dose-dependent manner after the experimental period (Figure 3A). The positive control group (FPC) rats presented the greatest levels of AFP, which significantly differed from the remaining groups. Figure 3B shows the CA15-3 values of the various experimental groups. Compared with all other groups, the FPC group presented the highest CA15-3 level, except for the rats administered a high dose of E155 (FHD), which was not significantly different. The rats administered E155 for feeding were significantly different from the control rats (FCs).

Histopathological analysis

Mammary Tumors

**Figure 4:**
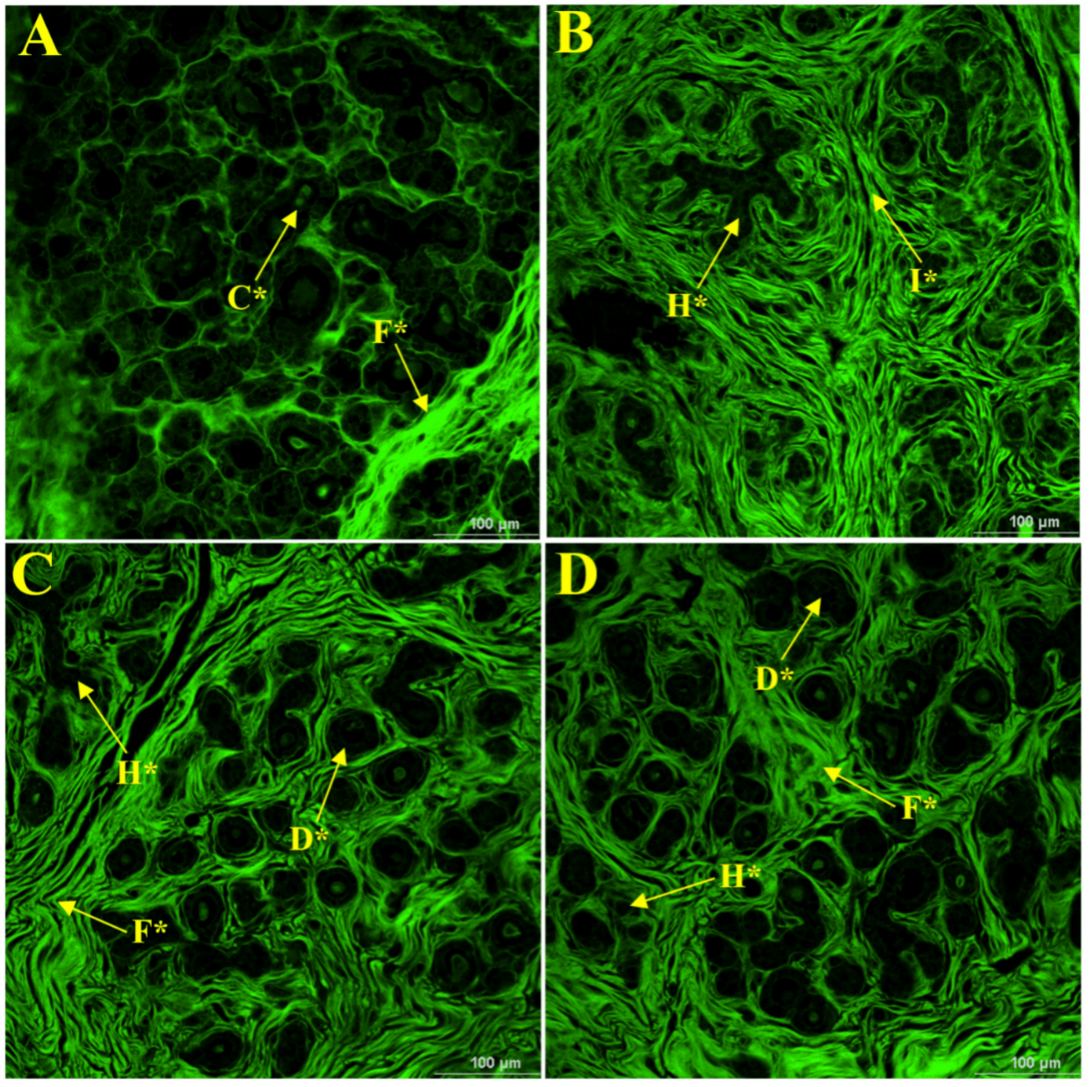
Investigation of the alterations in the histopathological structure of mammary tumors induced by brown HT (E155) in virgin female Long-Evans rats. Visualization of an object 40× and a 100-μm scale bar via a confocal microscope (model: Ti2-E Nikon). Mammary tumors in female positive control rats treated with the carcinogen DMBA at a dose of 3 mg/kg body weight (A), brown HT (E155) at a dose of 200 mg/kg body weight (B), brown HT (E155) at a dose of 400 mg/kg body weight (C), and brown HT (E155) at a dose of 600 mg/kg body weight (D). C* indicates carcinoma, D* indicates ductal carcinoma in situ, F* indicates fibroadenomas, H* indicates hyperplasia, and I* indicates infiltrative growth DMBA: dimethylbenz(a)anthracene

In this study, histopathological assessment of mammary tumors from female Long-Evans rats treated with different concentrations of brown HT (E155) shed light on its probable carcinogenicity (Figure 4). The positive control group (FPC) (Figure 4A), which was not treated with brown HT but received DMBA orally at a dose of 3 mg/kg BW, presented various histopathological lesions. These included carcinomas (C*), fibroadenomas (F*), ductal carcinoma in situ (DCIS), and hyperplasia (H*). The combination of fibroadenomas and hyperplasia results in diverse tumorigenesis responses in mammary tissues under these experimental conditions. In the group exposed to 200 mg/kg body weight brown HT (Figure 4B), histopathological analysis revealed the presence of hyperplasia (H*) and infiltrative growth (I*). Administration of a dose of 400 mg/kg BW (Figure 4C) resulted in the presence of fibroadenomas (F*), DCIS, and hyperplasia (H*). Similar histopathological changes were observed in the FMD group (Figure 4C) but were more severe in the FHD group (Figure 4D), which included animals treated with the highest dose (600 mg/kg BW), fibroadenomas (F*), DCIS, and hyperplasia (H*). Importantly, lesions tended to grow increasingly severely and frequently at this higher dosage, providing further evidence of the dose-dependent progression of the effects of brown HT on mammary tissue.

Small Intestine

**Figure 5:**
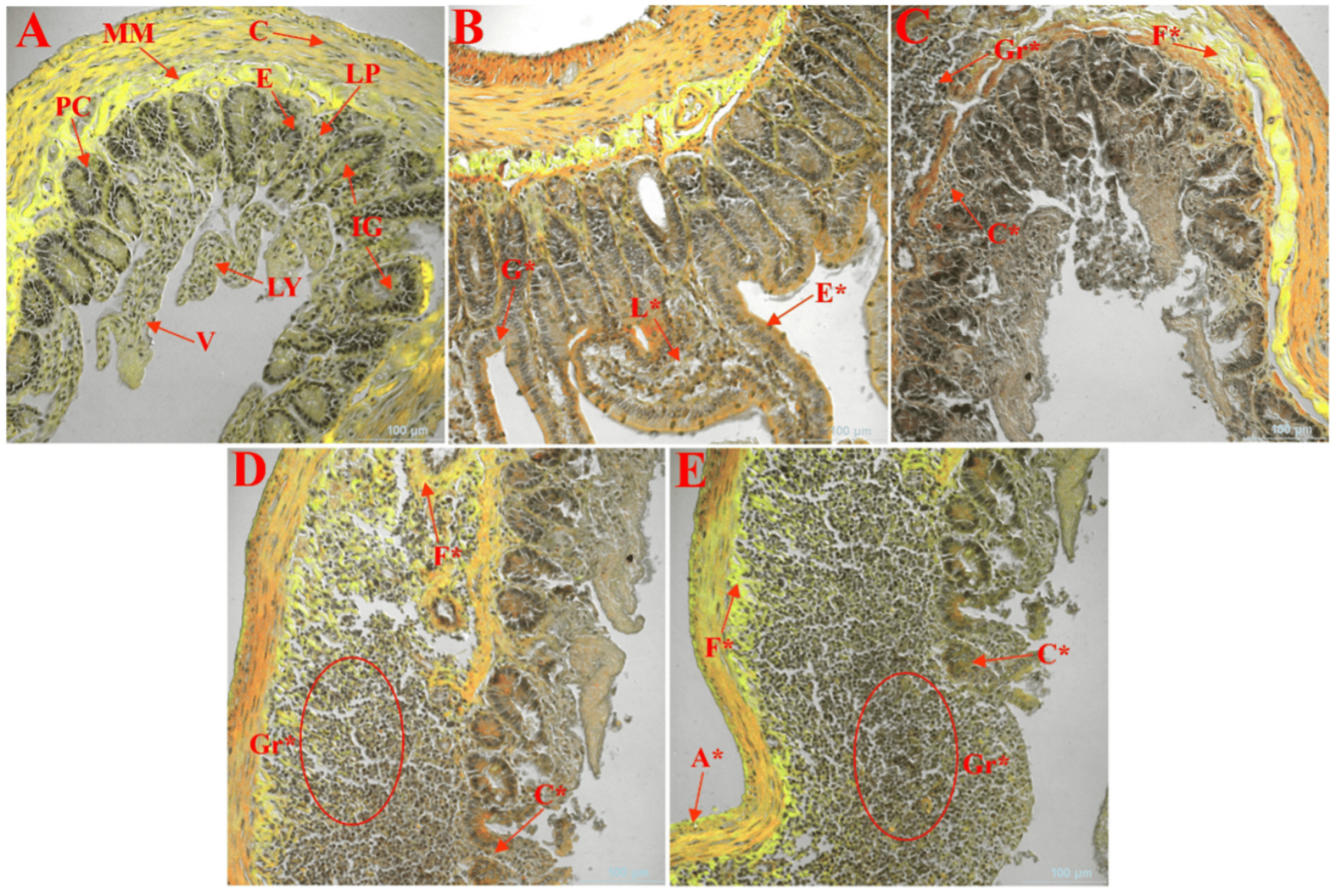
Investigation of the alterations in the histopathological structure of the small intestine induced by brown HT (E155) in virgin female Long-Evans rats. Visualization of an object 40× and a 100-μm scale bar via a confocal microscope (model: Ti2-E Nikon). A section of the small intestine is shown: control female rats that were not given brown HT as part of their diet (A), brown HT (E155) at a dose of 200 mg/kg body weight (B), brown HT (E155) at a dose of 400 mg/kg body weight (C), brown HT (E-155) at a dose of 600 mg/kg body weight (D), and female positive control rats treated with the carcinogen DMBA at a dose of 3 mg/kg body weight (E). A* indicates atrophy of the longitudinal, C indicates circular “inner” layer of muscularis externa, C* indicates crypt abscesses, E indicates enteroendocrine, E* indicates enterocyte hyperplasia, F* indicates fibrosis in the circular layer, G* indicates goblet cell depletion, Gr* indicates granulomas, IG indicates intestinal glands “crypts,” L* indicates lamina propina edema, LP indicates lamina propria, LY indicates lymphocytes, MM indicates muscularis mucosae, PC indicates Paneth cells, and V indicates villi DMBA: dimethylbenz(a)anthracene

As shown in Figure 5, the normal intestinal structure of the female rats in the control group FC (Figure 5A) was observed, as these rats did not consume brown HT as part of their diet. These include the circular outer layer of the muscularis externa, enteroendocrine cells, lamina propria (LP), LY, muscularis mucosa, Paneth cells, and villi. The observations revealed a normal, uncompromised state of the small intestine, which served as a control for the treatment groups. The group that was administered 200 mg/kg BW brown HT (Figure 5B) presented considerable pathological characteristics. These included goblet cell deficiency (G*), LP swelling (L*), and enterocyte hyperplasia (E*). Rats that received 400 mg/kg BW brown HT (Figure 5C) showed more pathological changes. Granulomas (Gr*), fibrosis in the circular layer (F*), and crypt abscesses (C*) were noted. Histology of the highest dose group receiving 600 mg/kg BW brown HT (Figure 5D) also revealed granulomas (Gr*), fibrosis of the circular layer (F*), and crypt abscesses (C*). The positive control group (Figure 5E), which received oral administration of the carcinogen DMBA at a dose of 3 mg/kg body mass, presented the worst intestinal lesions. Granulomas (Gr*), fibrosis in the circular layer (F*), crypt abscesses (C*), and atrophy of the longitudinal layer (A*) were noted. These pathological alterations support the carcinogenic effect of DMBA, which provokes inflammatory and neoplastic processes in the GI tract.

## Discussion

Breast cancer is the most commonly observed form of cancer among women, with approximately 2.3 million new cases reported worldwide each year [23]. The DMBA-induced mammary tumor model in rats has various characteristics that render it valuable for the investigation of human breast cancer. Upon metabolic activation by the cytochrome P450 enzyme, DMBA transforms into the ultimate carcinogen DMBA-3,4-dihydrodiol-1,2-epoxide. This metabolic activity generates different ROS, leading to the disruption of tissue redox equilibrium. These reactive species promote excessive production of lipid peroxidation, specifically in the form of malondialdehyde (MDA). Elevated levels of MDA have been extensively recognized in animal models and human cancer patients [24,25]. Nutritional and dietary components are crucial factors in the development of breast cancer. Several food additives have been recognized for their ability to cause toxicity in specific bodily processes. Many food industries use synthetic azo dyes, which, when ingested, can cause certain types of cancer owing to their cleavage byproducts [4].

The results of our investigation indicate that the ingestion of the azo dye E155 leads to the development of tumors and to a decrease in the rate of BW gain over a specific period of time, which is consistent with findings from prior research [26]. The observed pattern of weight loss or decreased weight growth is an indication of toxicity. Tumors were the most prevalent in the DMBA-induced FPC group. When the rats were administered a high dose of E155, they developed 18 tumors similar to those in the positive control group. Although the tumor volume in the FPC group was the greatest, it was not significantly different from that in the E155 treatment groups. These findings suggest that brown HT plays a role in the initiation and progression of mammary tumors, potentially leading to breast cancer. Zingue et al. reported that the presence of azo dyes leads to a greater occurrence of mammary tumors in rats [9].

Complete blood count indices are useful indicators of the degree of injury caused by a particular chemical. The data revealed that E155 significantly affected the RBC, Hb, MCV, MCH, and MCHC levels. Tomita et al. observed that hemoglobin levels ranged from 12.4 to 14.1 g/dL or lower, and MCV ranged from 43.3 to 49.3 fL or below, indicative of microcytic anemia, consistent with our findings [15,16]. This indicates that constant consumption of E155 results in microcytic anemia. Anemia may be due to the inhibition of erythropoiesis in the marrow, as suggested previously. The platelet count increased in a dose-dependent manner, potentially because of secondary thrombocytosis. Platelets serve as moving cellular sensors that provide a distinct connection between immunological reactions and tissue repair [27]. Elevated platelets occur because of the immune system's response to infection, inflammation, tissue damage, and tumors [28]. This was apparent from the increased numbers of neutrophils, monocytes, eosinophils, and basophils in the treatment groups. These results corroborate the reported tissue damage in the examined organs, including degeneration and inflammation, indicating that the immune system has been stimulated to enhance WBC production.

As in previous studies, alterations in the lipid profiles of rats fed E155 were evident [29]. Based on this investigation, it can be postulated that this dye may accentuate hepatic dysfunction, thereby increasing serum cholesterol levels. It also led to a significant elevation in SGOT and SGPT levels. This result supports a prior investigation [28]. SGPT and SGOT are considered highly specific biomarkers for determining liver function and detecting liver cell damage [30]. Elevated serum aminotransferase levels could be partly due to hepatic, renal, or cardiac parenchyma injury. According to another study, the level and severity of damage resulting from consuming toxic substances increase the presence of high levels of certain enzymes in the bloodstream [31]. A striking increase in bilirubin levels was observed, which can be explained by hemolytic processes and hepatic damage. Liver damage may result from excessive generation of ROS by this azo dye during metabolic transformation in the body [10]. There was also an increase in the severity of blood creatinine and uric acid levels in the treatment groups, supporting the possibility that E155 may contribute to renal impairment.

This study aimed to determine the levels of AFP and CA15-3 with the aim of establishing the presence or absence of tumors and breast cancer. AFP is one of the most recognized oncofetal antigens and is a 69-kDa glycoprotein in fetal serum. Initially, it is produced in the yolk sac during early pregnancy, but later in pregnancy, it is produced by the liver. This technique is used to screen developing fetuses and detect tumors in the mother [32]. It plays a role in several processes that occur in the body, including the growth, differentiation, and survival of cells, such as embryonic and tumor cells [33]. CA15-3 can be considered the best biomarker to predict the outcome of patients with breast cancer since it reflects the progression, regression, or even status of the cancer in people [34]. This investigation revealed a significant increase in the mean AFP and CA15-3 levels in the E155-treated rats compared to those in the control group. The FHD group did not significantly differ in CA15-3 values from the positive control group, which was treated with DMBA. The group was distinguished by a greater number of tumors and an evident increase in tumor size. The high levels of AFP and CA15-3 observed in the current study are suggestive of cancer and are in line with the findings of a previous study [9]. The metabolic conversion of azo dyes to aromatic amines during prolonged administration may lead to oxidative stress, potentially contributing to malignancy.

Histopathological changes in the mammary tissue of the positive control group shown in Figure 4A, which received only DMBA, revealed a high rate of malignant changes, such as carcinoma and DCIS. These outcomes show the high potential of DMBA to act as an initiator of carcinogenic changes in addition to hyperplasia and atypical hyperplasia, which are early precancerous changes [35]. As observed in the FLD group treated with a low dose (200 mg/kg BW/day) of brown HT (Figure 4B), the histopathological profile changed from benign to malignant. Indeed, with the appearance of infiltrative growth, malignant mammary tumors commonly infiltrate surrounding tissues, exhibiting unclear tumor borders that blend with nearby structures. Additionally, hyperplasia of the mammary tissue in this group indicates early dysregulation of the tissue, which may lead to future malignant changes in the event that they continue to be exposed [4]. Similarly, an increase in the brown HT dosage aggravated the severity and frequency of histopathological alterations. The FMD group received 400 mg/kg BW/day brown HT and experienced significant progression to DCIS, which can be associated with the most severe cases of tissue dysregulation and is considered the cancer precursor stage, as shown in Figure 4C. The worst effects were observed in the FHD group (Figure 4D), which received the maximum dose of 600 mg/kg. This group presented with increased severity and frequency of fibroadenomas, DCIS, and hyperplasia, indicating the carcinogenicity of brown HT in a dose-dependent manner [36]. The results of this study correlate with and support earlier findings related to the cancer hazards posed by synthetic dyes, particularly in mammary tissues. In addition, the increased levels of tumor markers, such as AFP and CA 15-3, revealed simultaneously in other studies, demonstrate the ability of brown HT to cause malignant changes in mammary tissues. These biomarkers are important in the prognosis and diagnosis of breast cancer; therefore, they increase the clinical relevance of the observed histopathological alterations [32].

The small intestines of the rats from all treatment groups were compared to determine the effects mentioned in Figure 5. The histomorphology of the sections of the small intestine from animals in the control group (Figure 5A) was not altered, the mucosal layer was unimpaired, and well-developed villi and crypts were observed. The lack of inflammation or neoplastic changes in this group correlates with the overall histological composition of the normal healthy mammalian small intestine [37]. Pathological changes observed in the FLD group (200 mg/kg BW/day brown HT) included the absence of goblet cells, dilated LP, and increased thickness of submucosal enterocytes (Figure 5B). Goblet cells secrete mucin, which forms a protective coat in the gut to avoid being attacked by pathogens and/or being torn apart by peristaltic movement. The observed deficiency in goblet cells indicates that the mucosal barrier is affected, which makes an organism prone to inflammatory processes and injury to the intestinal tract. Additionally, inflammation of the LP and enterocyte hypertrophy can be considered signs of pathological changes in the initial stages [38]. These animals are shown in Figures 5C, 5D, where more pathological changes and developments, such as granulomas, fibrosis of the circular muscle layer, and crypt abscesses, were observed. Granulomas usually indicate chronic inflammation and diseases such as Crohn's disease, which means that there is a residual immunological response to the presence of brown HT. Notably, the fibrosis and crypt abscesses in these groups suggest persistent tissue remodeling and inflammation, leading to severe architectural alterations in the intestines. These lesions are more severe with increased doses of brown HT, suggesting a dose-dependent toxicological effect with the consumption of higher amounts of brown HT [1]. More severe intestinal lesions were observed in the FPC group (Figure 5E). From a histopathological perspective, the main gross findings were granulomas and apparent fibrosis, mainly on the circular layer wall, the presence of crypt abscesses, and atrophy of the longitudinal muscle layer. Atrophy of the longitudinal layer indicates severe pathogenic damage, potentially impairing nutrient absorption and overall GI function [1,38]. The general pathological findings of this study offer a definitive assessment of the dose-related and profound structural alterations observed in the intestinal region due to both brown HT and DMBA and implications for health in the future.

Limitations include external validity of findings from animal studies reflected in humans due to species-specific genetic variation in metabolism, dose relevance to reflect human exposure level, exposure duration, and frequency in the real world. In vitro, cell line models have been extensively used in fundamental cancer research. Further investigation is required to address these limitations and to unveil the molecular mechanism of chocolate brown HT (E155) in the etiology of mammary tumors. An in vitro cell line model is also recommended to enhance our understanding of the potential risk of breast cancer. Under optimal settings and with suitable controls, cancer cell lines preserve most of the genetic characteristics of their malignancy of origin.

## Conclusions

This study demonstrated that consumption of chocolate brown HT (E155), a commonly used azo dye, leads to the development and expansion of mammary tumors, even when consumed at low doses for an extended period of time. Increased levels of AFP and CA 15-3, especially at moderate and high doses, suggest a potential link to the development of breast cancer. When administered at a dose of 200 mg/kg BW/day, it caused an aberrant growth response and disrupted the structure of mammary tissue. Anomalous proliferation of mammary ducts was observed at moderate to high dosages. In addition, the presence of chronic inflammation was identified by analyzing the intestinal histology and hematological data. This dye causes macrocytic anemia as well as damage to the liver and kidneys. Due to the widespread and unregulated use of azo dyes in food products, policymakers need to stress this issue to adopt strict policy guidelines and enhance public awareness to limit the use of this dye. The findings from the current study may serve as a foundation for future research to thoroughly examine the mechanism of action of this dye in the etiology of these complications.
